# How Does Changing Environment Influence Plant Seed Movements as Populations of Dispersal Vectors Decline?

**DOI:** 10.3390/plants12071462

**Published:** 2023-03-27

**Authors:** Jonathan O. Hernandez, Muhammad Naeem, Wajid Zaman

**Affiliations:** 1 Department of Forest Biological Sciences, College of Forestry and Natural Resources, University of the Philippines Los Baños, Los Baños 4031, Laguna, Philippines; 2 Department of Plant Science, School of Agriculture and Biology, Shanghai Jiao Tong University, 800 Dongchuan Road, Shanghai 200240, China; 3 Department of Life Sciences, Yeungnam University, Gyeongsan 38541, Republic of Korea

**Keywords:** defaunation, habitat fragmentation, spore dispersal, propagule dispersal, PRISMA, zoochory

## Abstract

Plants differ widely in their ability to find tolerable climatic ranges through seed dispersal, depending on their life-history traits and habitat characteristics. Following the Preferred Reporting Items for Systematic Reviews and Meta-Analyses (PRISMA) guidelines, a systematic review on seed dispersal mechanisms was conducted to elucidate plant seed movements amid changing environments. Here, the highest relative count of studies was found in Spain (16.47%), followed by Brazil (14.12%), and the USA (14.12%). The megadiverse, hotspot countries (e.g., Philippines, Vietnam, Myanmar, India, and Indonesia) and Africa (Tanzania, South Africa, Democratic Republic of the Congo) have very low to no data about the reviewed topic. The effects of land use changes, habitat degradation/disturbances, climate, and extreme weather conditions on seed dispersal mechanisms and agents had the highest share of studies across topics and countries. Plant diversity and distribution of anemochorous, endozoochorous, epizoochorous, hydrochorous, myrmecochorous, and ornithochorous species are seriously affected by changing environments due to altered long-distance seed dispersal. The fruit types commonly associated with endozoochory and ornithochory are species with achene, capsule, drupe, fleshy, and nut fruits/seeds, whereas achene, capsule, samara/winged seeds are associated with anemochory. The present review provides a summary of evidence on how plants are affected by climate change as populations of dispersal vectors decline. Finally, recommendations for further study were made based on the identified knowledge gaps.

## 1. Introduction

Climate change is contributing to the rapid range shift and loss of plants and animals towards higher elevations as they keep up with elevated atmospheric CO_2_ concentrations, a warming climate, and altered precipitation regimes [1,2]. Many species will have to relocate as the climate warms to function and survive according to temperature and precipitation ranges that they can tolerate. As plants are immobile, they are expected to differ widely in their ability to find climatic ranges they can tolerate, depending on their traits and habitat characteristics [3]. One way to explain how disperser species range shifts and track climate change is by quantifying species dispersal ability based on dispersal-related traits (e.g., vectors, seed mass, seed/fruit types).

Seed dispersal, defined as the transport of seeds, spores, or propagules away from the mother plant, is one of the key processes in ecology and biodiversity conservation. Dispersion of seeds is the first spatial demographic process that can determine potential range shifts [4]. It is also a critical factor driving plant ecological responses to global environmental changes [5]. Environmental changes can disrupt the seed dispersal process, and such a disruption might either increase or decrease dispersal distance depending on the dispersal mode of the species [6]. For example, anemochorous seeds (wind-dispersed) can travel much further in open or disturbed landscapes than in dense or undisturbed forests because of contrasting wind speed, direction, and intensity [7]. Thus, any type of severe habitat disturbance will significantly alter the patterns in seed movement, recruitment, and establishment of plant populations [8].

However, the literature on seed dispersal is focused on the dispersal syndrome hypothesis (DSH) or co-adapted syndrome, which states that fruits and dispersal vectors co-evolved (mutualistic interactions) influencing the foraging strategies of dispersers (e.g., [9]). A large amount of information focuses on only one environmental change driver (e.g., habitat fragmentation) affecting the seed dispersal processes (e.g., [10]). Although there are overwhelming theoretical studies on the enumeration or assessment of dispersal vectors of a particular group of plants (e.g., [11,12,13]), information on how the dispersal mechanisms of many plants evolve in a changing environment remains scarce. While studies on how global environmental drivers and their interaction affect plant movement have considerably received much attention [6,14], the mechanisms by which changing environmental conditions affect seed transport between sites are still unclear because of contrasting results. These contrasting results are attributed to geographical locations, species life-history traits, variability in seed dispersal-related traits, habitat structure, and complex mutualistic interactions. The seed dispersal capacities of many species are also not known, which limits our understanding of the seed dispersal disruption caused by global change drivers. Moreover, studying how plant populations track their niche through seed dispersal in a changing environment is also empirically difficult to study due to logistical and technical challenges [15].

Consequently, a systematic review of the current state of the literature about seed dispersal mechanisms in a changing environment was conducted to help us understand how plants keep up with climate change as populations of dispersal vectors decline. The obtained literature was summarized and analyzed to give insight into (1) the geographical distribution of studies about seed dispersal in a changing environment; (2) how the changing environment affects seed dispersal; (3) whether changing environment reduces or enhances plants dispersal abilities; (4) seed/fruit dispersal-related traits, and (5) research directions. The present review is relevant to understanding plant seed movements in the face of anthropogenic climate change.

## 2. Results

**Figure 1:**
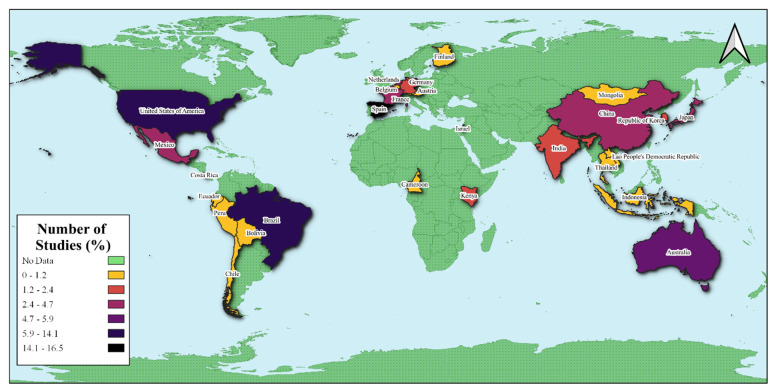
Geographical distributions of the reviewed studies on seed dispersal mechanisms in a changing environment.

The literature search returned 10,651 articles from three databases (ScienceDirect, PubMed, and Google Scholar) and direct search. After the selection process, only 118 peer-reviewed articles were left for further analysis. The present systematic review found the highest number of studies in Spain (16.47%), followed by Brazil (14.12%), and the USA (14.12%) (Figure 1). Australia, China, Israel, and the Netherlands had only 4.71–5.88% relative count of studies. Most of the megadiverse countries in Asia (e.g., Philippines, Vietnam, Myanmar, India, and Indonesia) and Africa (Tanzania, South Africa, Democratic Republic of the Congo) have very low to no data about the reviewed topic.

**Figure 2:**
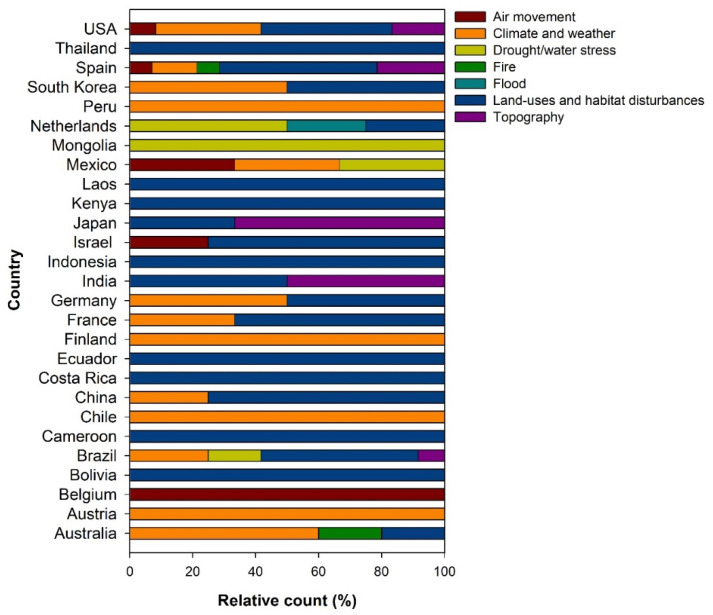
Frequently studied topics about seed dispersal mechanisms in a changing environment across countries.

The effects of land use changes, habitat degradation/disturbances, climate, and weather on seed dispersal had the highest share of studies across topics and countries (Figure 2). Under land use changes, the effects of urbanization, forest or grassland conversion into agricultural lands, and any forms of natural landscape modifications on seed dispersal mechanisms, were frequently studied. The topics identified under habitat degradation/disturbance include human-induced habitat fragmentation, deforestation, runoff and erosion, hunting/harvesting pressure, and defaunation. Topics such as seed dispersal in heterogeneous environments and changing landscapes were also frequently studied. Studies on the effects of climate change, rainfall and temperature gradients, and extreme weather conditions on seed dispersal modes and agents for various groups of plants were also common in many countries.

Studies about the effects of topography (e.g., elevation gradient and slope), drought/water stress, and air movement (e.g., speed and direction) ranked second in the relative count of topics. Natural fires and floods and seed dispersal patterns had the lowest relative count of studies across countries.

Results of the present systematic review revealed the most and least frequently studied dispersal modes depending on fruit type (Table 1). Here, the endozoochory seed dispersal had the highest relative count (33.33%), followed by ornithochory (22.58%), and anemochory (17.21%). The other identified dispersal modes that are affected by the changing environment include hydrochory, myrmecochory, epizoochory, and autochory/ballochory, with less than 10% relative count of studies.

In the reviewed studies, the commonly associated fruit types with endozoochory or animal-mediated seed dispersal are species with achene, capsule, drupe, fleshy, and nut fruits/seeds, such as those in *Tristerix corymbosus*, *Ficus carica*, *Duckeodendron cestroides*. Ornithochorous (bird-dispersed) and endozoochorous species typically have the same fruit types. Contrarily, fruits or seeds of wind-dispersed species (e.g., *Erigeron bonariensis*, *Pinus halepensis*, *Swietenia humilis*) are generally achene, capsule, or samara/winged.

Table 2 shows the morpho-structural, biochemical, and molecular traits related to short or long-distance seed dispersal modes of different plants. Depending on pericarp consistency, fleshy fruits may be grouped into different types, such as berry, drupe, hesperidium, pepo, and pome. Dry fruits consist of two major classifications, the dehiscent (capsule, silique, follicle, and legume) which splits open when mature, and indehiscent (caryopsis, nut/acorn, samara, achene), which do not split open at maturity. All the morpho-structural, biochemical, and molecular traits allow efficient and effective seed dispersal depending on dispersal vectors.

**Figure 3:**
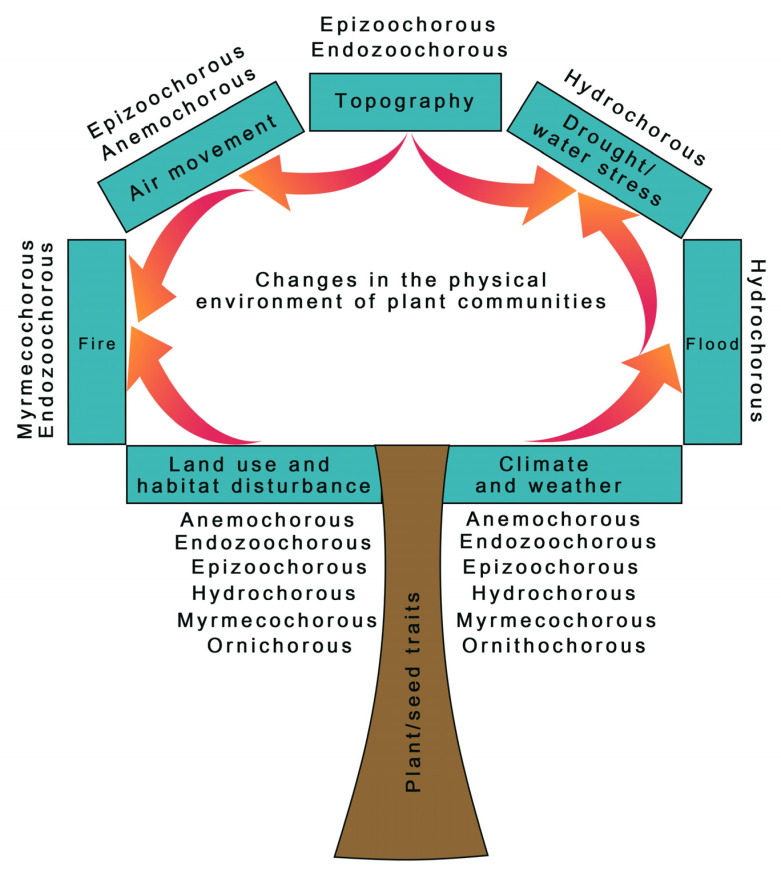
Diagram of seven environmental factors influencing seed dispersal modes of different plant groups. The arrows represent the interactions among factors.

Overall, the seed dispersal modes as affected by the ever-changing environment are summarized in Figure 3. Our systematic review revealed that significant changes in land uses, habitats, and environmental conditions, particularly climate and weather, may pose serious threats to at least six groups of plants, namely anemochorous, endozoochorous, epizoochorous, hydrochorous, myrmecochorous, and ornithohorous species. It was also found that changes in the speed and direction of air, elevation, and slope could also affect the dispersal of seeds depending on their fruit types of dispersal modes (e.g., anemochorous species for changes in air movement). Natural disturbances, such as drought, fire, and flood, and their interactions, also pose significant modifications in the manner how seeds of a particular group of plants are dispersed in an area. For example, prolonged drought and fire could engender a significant threat to hydrochorous and myrmecochorous plant species, respectively.

## 3. Discussion

### 3.1. Geographical Distribution of Studies on Seed Dispersal in a Changing Environment

Seed dispersal is critical for plant population recruitment, gene flow, long-term survival, and other ecological processes amid climate change [24,25]. This has recently raised the priority of plant seed dispersal research in many countries. However, the present systematic review found very little to no data on seed dispersal research in several megadiverse and hotspot countries, suggesting that the reviewed topic has hardly been studied in many key biodiversity areas in the world. As global climate change is predicted to have major effects on species’ distributions [25], seed dispersal research in megadiverse and hotspot countries should be enhanced to promote high species diversity. This is because the manner in how the seeds are dispersed from the mother plants determines the recruitment capacity, which is a prerequisite of plant community establishment and development [26]. Knowledge of seed dispersal can benefit the development of effective management and conservation options for threatened species in megadiverse and hotspot countries. Seed dispersal studies in these countries will help elucidate the temporal and spatial patterns of plant movements as well as behavioral mechanisms of dispersal agents in plant communities following global change. Field-based seed dispersal studies in megadiverse and hotspot countries will also be able to assess currently important biotic dispersal vectors for conservation as most plant species depend on animals and fungi to disperse their seeds [27,28,29]. The overhunting of ecologically important vertebrates, for instance, can cause vertebrate-mediated tree species to suffer from reduced seed dispersal [30] and impoverished seedling communities in terms of diversity and seed size [31]. Many of the hunted species are frugivores, which are major dispersers of tree and shrub seeds [32].

### 3.2. How Does Changing Environment Affect Seed Dispersal?

**Figure 4:**
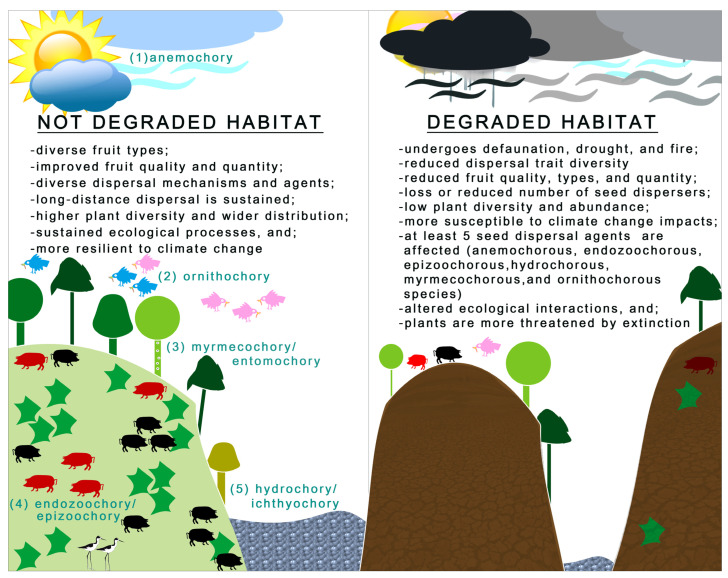
Contrasting seed dispersal modes and agents between not degraded and degraded habitats.

Here, land use changes, habitat degradation/disturbances, and climate and extreme weather conditions have a significant influence on seed dispersal mechanisms of various groups of plants across different ecosystems in temperate and tropical regions (Figure 4) [33,34,35,36]. Previous studies have shown significant increases in dispersal trait diversity and relative abundance of biotically dispersed seeds over time in vacant lots with varying land use histories and non-urban grassland habitats [37,38,39]. Although it has been suggested that seed dispersal is a significant driver of urban plant community assembly patterns, urbanization has a severe influence on many native epizoochorous species acting as dispersal agents of native plants [37,40]. While urban sites had three times more disperser species when compared to natural habitats, dispersal success or germination rate was significantly lower in urban sites, resulting in an overall negative effect on the seed dispersal of *Toxicodendron radicans* (L.) Kuntze [40,41].

Increasing habitat fragmentation has shown direct and indirect effects on seed dispersal patterns, including the negative effect of connectivity on seed dispersal potential and reduced dispersal abundance [42,43,44]. Habit degradation affects habitat quality and heterogeneity; thus, it largely influences seed deposition due to reduced vegetation cover and fruit supply for bird dispersers [45]. Habitat quality is positively associated with seed dispersal distance, suggesting that the dispersal patterns can be predicted by habitat characteristics [46]. In the tropics, the number and distributions of dispersed seeds were all reduced in fragmented habitats, particularly for large animal-mediated plants [47]. In a fragmented subtropical rainforest, large-seeded plants are also vulnerable to limited dispersal [48], and this is consistent with the findings found in hunted or selectively logged forests [49]. A similar pattern was observed in a fragmented forest in Spain, such that the likelihood of seed deposition decreased significantly because there were fewer areas with trees for perches and fruit for bird dispersers [50]. Even in an arid and semi-arid country, seed dispersal rates were significantly lower in disturbed forest habitats than in undisturbed ones, and the result was also attributed to a reduced abundance of frugivores or seed dispersers [51]. A study conducted in a disturbed habitat in the Mediterranean Basin yielded a similar result, i.e., a reduction in habitat complexity negatively impacted bird abundance due to altered fruit movement and simplification of the seed shadows [52]. Seed dispersal was also two-fold higher at the protected than at the unprotected sites, even though both sites have been selectively logged in the past [53]. The authors attributed the findings to changes in activity patterns, quantity, or population makeup of vertebrate seed eaters between sites, which had been subjected to the unsustainable hunting of terrestrial mammal dispersers.

The fate of biodiversity will be heavily influenced by species’ ability to adapt to climate change, yet very little is known about how many species will disperse in a changing environment [54]. A model showed only species with the highest climatic suitability and dispersal capacity are expected to survive climate change [55]. Ever-changing climatic patterns influence various ecological processes, including seed dispersal and animal populations acting as important seed dispersers [56,57,58]. A model revealed that various scenarios for climate change predicted varying degrees of phenological mismatch between plants and the birds that disperse seeds, leading to major declines in germination success [59] and, thus, biodiversity outcomes [60]. Specifically, climate-change-induced defaunation (decline in animal populations) has severely reduced long-distance dispersal (LDD) by nearly half the number of seeds dispersed based on trait-based models [61]. In the Amazon rainforest, a forecast study showed that seed dispersal might be hampered by climate change due to niche mismatch and disruption of biotic interactions, even if defaunation is controlled [62]. Mismatches between population niches and geographic distributions are most pronounced in poorly dispersed and extremely persistent plant species subjected to severe environmental change [63]. In terms of the effect of rainfall on seed dispersal patterns, a study indicated that an increase in average annual rainfall in Brazil was related to a gradual increase in the percentage of vertebrate-dispersed species, particularly berry species [64]. A similar result was reported in China, i.e., mean annual precipitation had the greatest predictive contribution to the dispersal of woody fleshy-fruited species in wet, warm, and stable environments [65]. It has also been reported that dispersal distances by rain were facilitated by increasing slope and decreasing seed volume, indicating that rainfall could be an important dispersal mode for small-seeded plants along an elevation gradient [66]. In the Atlantic Forest, elevation and rainfall had no interacting effect on the seed distribution of endozoochorous species, but a main effect of annual mean rainfall was observed, particularly in moister areas [67]. Overall, the results presented above provide evidence that global climate changes may negatively affect the seed dispersal patterns of vertebrate-dispersed plants, thereby determining the future of biodiversity.

### 3.3. Will the Changing Environment Reduce or Enhance Plants’ Dispersal Abilities?

The impacts of climate change directly and indirectly affect seed dispersal by altering the biophysical environment (e.g., habitat quality, fruit availability, phenology) and plant/seed traits by means of temperature, rainfall, wind speed, windstorms, etc. [68]. According to the results of the present review, at least six groups of plant species are seriously impacted by changing environments (Figure 4). Specifically, increases in air temperature can improve the long-distance seed dispersal of anemochorous species [69], and significant changes in wind speed determine their dispersal rates [70]. An analytical model predicted that seed dispersal and potential population spread rates of anemochorous species should decrease with reductions in wind speed [71]. The authors further noted that dispersal of the wind-dispersed seeds varied between native and non-native species. Approximately 500 species of anemochorous species widely distributed in Asian tropical forests generally have bract-winged samaras, whose long-distance dispersal is affected not only by morphological traits (e.g., seed mass) but also by environmental factors [12,72]. Thus, anemochorous species that rely on wind for long-distance seed dispersal may be vulnerable to the impacts of climate change-driven changes in wind patterns.

Dispersal potential is one of the factors influencing the migration of plant species [73]. This highlights the significance of investigating vertebrate-mediated seed dispersal, which is one of the most efficient modes of long-distance dispersal for many plant species [74]. Here, evidence shows that changing plant environments, particularly climate, can pose serious threats to animal-dispersed trees (e.g., endozoochorous, epizoochorous, and ornithochorous) due to dispersal limitations. Such limitations can be due to climate change-induced reduction in populations of animal dispersers, particularly large-bodied and threatened species, and changes in feeding habits [75,76]. Analysis shows that an average of 18% of mammal, bird, and amphibian species have lost their natural range sizes due to global changes [77]. In north-eastern Brazil, a low rainfall gradient had a significantly lower rate of vertebrate-dispersed species than in a high rainfall gradient [64]. Low-rainfall areas are not suitable habitats for large fleshy fruited species, which are preferred by frugivores [64], and as a result, the diversity of endozoochorous tree species may drop [78]. Contrarily, the richness of fleshy-fruited species was found to be greater in places with low soil water content but at lower elevations than in higher-elevation sites [18]. The authors ascribed the findings to high temperatures, which can improve plant water availability, and low evapotranspiration in low elevations, allowing for more water to be available to produce fleshy fruits. Fleshy fruits with high water content are more attractive to animal dispersers [18].

Ornithochorous species also aid plant species in adapting to climate change through long-distance seed dispersal, although bird migration to disperse seeds is potentially limited to cooler latitudes [79]. The authors also found that migratory birds disperse seeds in the wrong direction to help keep track of climate change. Specifically, mean annual precipitation was found as a significant driver for bird species richness in arid and semiarid regions [80]. Increasing precipitation and temperature patterns may result in declines in birds due to the disruption of their habitat and food resource by flooding [81,82,83] and birds’ geographic ranges and migration phenology [84]. A study also reported that increasing drought may negatively affect bird populations in the Bahamas due to annual variations in rainfall and habitat quality [85]. Moreover, fruit-eating bird dispersers vary in terms of mobility, habitat requirements, food-foraging techniques, and digestive capacity, all of which affect the quantity of seeds that may be dispersed [86]. Thus, climate change-induced loss of birds could strongly influence the establishment or regeneration dynamics of ornithochorous plant communities by curbing seed dispersal.

Hydrochory (seed dispersal by water), which is common in low-lying areas that are frequently flooded, is one of the adaptations of plants in changing environments [87,88]. The current systematic review found that a substantial proportion of plant species use water for dispersal or seed transport, which has also evolved to adapt to changing environmental conditions (e.g., flood and drought). In a river valley in Germany, hydrochorous dispersal processes were found to be beneficial in connecting fragmented populations of water-mediated plants in regularly flooded riparian habitats [89]. Contrarily, some hydrochorous seeds can be destroyed by fish (facultative ichthychory) in the seasonally inundated floodplain forests of Amazonia instead of dispersing the seeds by water [90]. Although flooding events can be an important means for seed dispersal to floodplain habitats, prolonged flooding can decrease seed viability, except for flood-tolerant hydrochorous species [91]. Some seeds may also break their dormancy while still submerged in flood water, resulting in risky underwater germination, and this can cause erratic germination and establishment in the floodplains [90,92]. Overall, alterations in duration, intensity, timing, and frequency of flooding or water level fluctuations can either disrupt or enhance the dispersal patterns of hydrochorous plant species.

Ants are one of the dominant terrestrial organisms with high seed dispersal capacities through myrmecochory, which also plays an important role in shaping plant communities. Climatic warming, however, alters ant–plant seed dispersal mutualism [93], thereby affecting the abundance and diversity of myrmecochorous species (e.g., *E. tetrodonta*). Myrmecochory seed dispersal, which involves ants collecting and transporting seeds with elaiosomes (rich in lipids and proteins found in fleshy fruits that are attached to the seeds), is a dominant dispersal mode in sclerophyll habitats [94]. These habitats are generally dominated by plants adapted to long periods of dryness and heat, and thus, can be prone to climate change-induced fire, which also influences ant abundance, for example fire-altered seed dispersal distance in the Australian tropical savanna [95]. Thus, climate-change-induced modifications in ant abundance, diversity, occurrence patterns, and interspecific interactions will have a significant impact on the structure and functioning of myrmecochorous plants [95,96], particularly in sclerophyll habitats.

### 3.4. Do Fruit Traits Control Long-Distance Seed Dispersal in a Changing Environment?

Seed dispersal responses to changing environmental conditions can be influenced by plant traits, specifically fruit and seed morphological and biochemical characteristics. Studies have suggested that traits such as fruit or seed size, color and odor influence the way dispersers choose them as food [16], thereby shaping plant distribution. Here, results revealed a large proportion of studies that mentioned achene, capsule, drupe, fleshy, and nuts as fruit/seed types of many endozoochorous and ornithochorous plant species. In a rainforest, the distribution patterns of *Endiandra* species revealed that large-fruited species had lower geographical ranges than small-fruited species [97]. Such a pattern was ascribed to the restricted presence of vertebrates that can ingest and disperse larger fruits due to differences in the intensity of temporal climatic fluctuations [97,98]. Secondary metabolites, which are usually accumulated at high levels in fleshy fruits, are also involved in the interaction of fleshy fruits with vertebrates, insects, and microorganisms seed dispersers [99]. Moreover, fleshy fruits are more colorful, particularly those trees and shrubs in forests, to attract dispersal agents and increase the chances of being seen by potential seed dispersers [100,101]. This exemplifies the mutualistic relationship between traits of fleshy fruits and their seed dispersers.

Mutualistic seed-dispersal relationships exist also between a range of nut-producing plants and animals (e.g., rodents) that scatter their nuts in the soil [102]. However, concerns have arisen about the ability of nut-bearing plants (e.g., oaks) to colonize new places or migrate in response to climate change due to their recognized dispersal constraint [103]. Rodents, which are one of the major dispersers of acorns (true nuts), generally do not prefer or cross open spaces [104] typical of degraded and fragmented habitats. Moreover, a study also showed sustained rodent population decline with climate warming [105].

Mature, dehiscent fruits, such as capsule, silique, follicle, and legume, have dead pericarp that is dry and hard, which helps in long-term storage of biologically active proteins, nutritional elements (e.g., nitrate, potassium, and phosphorus), and other substances [22]. These substances may influence fruit/seed size, shape, texture, composition, and dehiscence property, which may indirectly influence the diversity of seed dispersal modes and distribution. The morphological and physiological characteristics of seeds or spores play important roles in determining the fate of seeds in unpredictable habitats [23]. Further, the dehiscence characteristic may help plants escape extreme environmental conditions through long-distance dispersal [106].

Indehiscent achenes are also dispersed by animals through adherence to the body of the dispersers. Hence, different sizes and structures of achenes can influence their dispersal over long distances. For example, the pappus geometry of achenes affects the aerodynamic properties of the seeds and responses to turbulence and humidity, which influence seed dispersal efficiency [107]. A study also found that the most successful colonizers, which generally produce achene fruits and/or seeds with pappus appendages, seemed to prefer warmer temperatures [108]. Moreover, dispersal rates of wind-dispersed achenes of *Senecio* species varied significantly with changes in the site, surroundings, height, time of release, direction, and achene type, indicating that distances of dispersal can be influenced by local conditions of humidity, wind, and vegetation structure [109].

### 3.5. Future Perspectives

The effects of environmental changes were largely unrepresented in some seed dispersal vectors (myrmecochory, hydrochory and ichthyochory, epizochory, and entomochory), which can be attributed to geographical, technical, and logistical constraints. For example, there are fewer scientists specializing in ants or insects in general compared with other research fields. Studying seed dispersal ecology (for example, forecasting dispersal processes) is also difficult due to seed dispersal dependence, environmental heterogeneity, and interdependent processes operating at various spatial and temporal scales [110]. More research investments in megadiverse and hotspot countries are needed for a better understanding of how plants can keep up with climate change, particularly the less-studied seed dispersal vectors. There is still a need for investigations that advance interdisciplinary perspectives and collaboration to study seed dispersal processes amid climate change. Integration of dispersal mechanisms of plant species in ecological studies would help us effectively predict biodiversity responses to global changes. Improvement of seed dispersal studies can help conservationists predict or prioritize areas and species for conservation [111]. Moreover, integrative research on the interacting effects of various extrinsic (e.g., the interaction of climate, drought, and habitat fragmentation) and intrinsic factors on seed dispersal processes across landscapes, plant growth forms, and taxa of dispersers is also promising. Significant advances in modelling the effects of fruit types on the seed dispersal ability of species should also be done to better understand a diverse array of dispersal strategies across taxa. Finally, all the recommendations made will be crucial for the evidence-based conservation of biodiversity and informed policy formulation amid changing environments.

## 4. Methodology

### 4.1. Literature Search and Data Sources

A systematic literature review (SLR) was performed from September to December 2022 to collect relevant literature on seed dispersal processes in a changing climate, and included all records published between 1990 and 2022. Following the literature search strategy typically utilized in biological science research, the SLR returned an initial total of 10,651 articles from ScienceDirect, PubMed, and Google Scholar search databases. These databases are among the most prominent peer-reviewed original article search engines, and they are frequently cited in published SLR articles across disciplines [112]. Some articles were retrieved by running a direct search on Google using the list of references/bibliography of the downloaded papers using the keyword “dispersal” through the find text function (Ctrl + F).

First, a search test was run to narrow the search keywords. The final search phrases were constructed, with the most significant keywords in each set of search terms, namely seed dispersal, spore dispersal, propagule dispersal, climate change, and changing climate (Table 3). Boolean search operators (i.e., “AND” and “OR”) were typed in all uppercase letters on the search tab of each database to exclude, broaden, or define the search results. The AND Boolean operator was used to include both important keywords (for example, “seed dispersal” AND “climate change”), while the OR operator was used to search for records in each database that contained any of the terms separated by the operator (for example “spore dispersal” OR “propagule dispersal”). Each keyword was enclosed by quotation marks (“”) to find the precise phrase or word. The advanced search tool of each database was employed by defining the keywords, publication year range, and article type. Too specific phrases (e.g., the specific name of a country, dispersal types, fruit types, etc.) were not included to minimize bias in the search terms.

### 4.2. Article Screening and Appraisal

**Figure 5:**
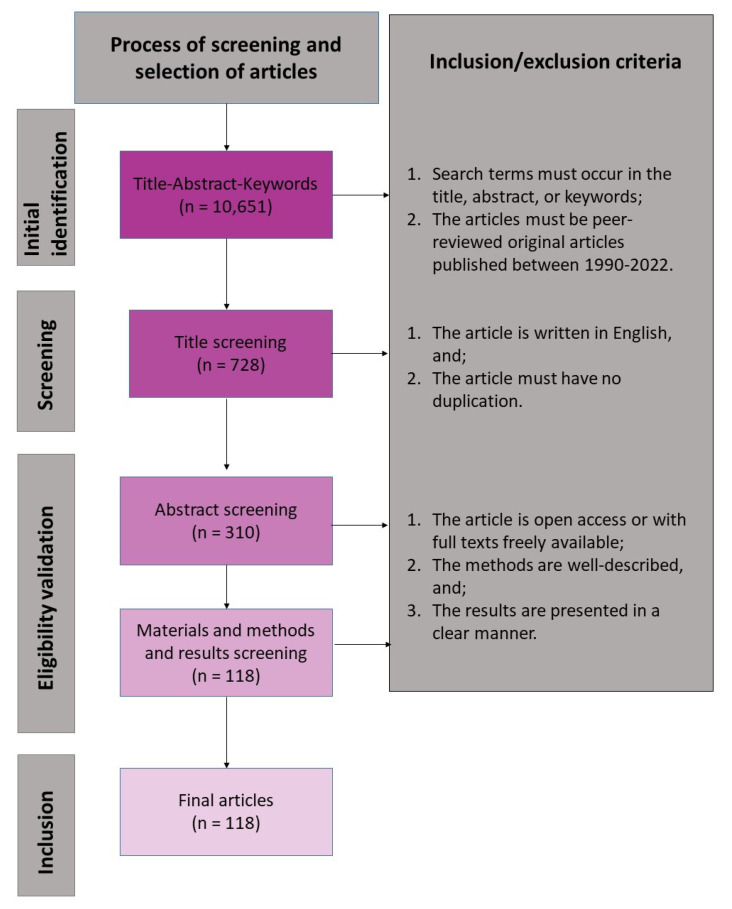
Preferred Reporting Items in Systematic Reviews and Meta-Analyses (PRISMA) flow diagram of inclusion and exclusion of peer-reviewed studies on seed dispersal in a changing environment that was used for the present systematic review. The n denotes the number of articles identified and included through a five-step selection process.

The articles were assessed for inclusion in the review using the Preferred Reporting Items for Systematic Reviews and Meta-Analyses (PRISMA) guidelines for article screening and appraisal. The screening procedure is shown in Figure 5, along with the inclusion and exclusion criteria. In this review, only one reviewer completed the screening to maintain uniformity and avoid selection mistakes and biases. To ensure a comprehensive representation of the literature, the SLR included all quantitative, qualitative, and a combination of the two methods that used either experimental, observational, or simulation approaches. The titles, abstracts, and keywords were reviewed first to exclude anything unrelated. In this step, search terms that did not appear in the paper’s title, abstract, or keywords were removed. The papers needed to have an emphasis on seed dispersal processes in a changing environment (e.g., climate, weather, habitat fragmentation, etc.). The SLR also excluded grey literature and articles that were not peer-reviewed and not published within the time frame specified. The papers were further filtered by removing articles that were not written in English-language journals, were irrelevant, or were duplicated. Using the Mendeley Reference Manager (version 2.72.0) and, in some cases, a pivot table in a Microsoft Excel Spreadsheet, articles with the same publication year, title, and author were removed at this stage. Based on an abstract skim reading, all publications that satisfied the initial set of inclusion criteria were chosen for additional research and content review. The SLR excluded articles that are not open access or have no free full texts. The databases provide links to PDF files of the publications, and if none, they were looked up on other research websites (e.g., ResearchGate). All abstracts were skim-read, and those with ambiguous conclusions were eliminated. Articles were validated by scanning the main text and concentrating primarily on the findings and methods portions of the publication. In this case, all papers with ambiguous results or that did not include original research data and no detailed explanation of the methods used were discarded.

### 4.3. Data Extraction, Categorization, and Analysis

Using the extraction criteria listed in Table 4, data were extracted from each article and encoded in Google Sheets. The year of publication was derived from the article’s page, and the study location (country) was derived from each article’s “Study site description”. All articles that had no information about the country were eliminated, and Google was used to search the country for articles that only included names of places (e.g., names of permanent plots). The research topic was principally generated from the keywords in the paper title, and it was then separated into seven groups: (1) air movement, (2) climate and weather, (3) drought/water stress, (4) fire, (5) flood, (6) land use and habitat degradation/disturbances, and (7topography. The effects of wind speed, intensity, and direction on seed dispersal patterns were the topics included in the first group. The second group focuses on the effects of changing climatic patterns (air temperature and rainfall) and extreme weather on seed dispersal. The sixth group is about the effects of land-use changes (e.g., grassland conversion) and human-induced habitat degradation/disturbances (e.g., deforestation, defaunation). The dominant plant species and their fruit types were also compiled to better explain their modes of dispersal. Most of the information on the dominant species and fruit types was provided by the experimental plant description in the materials and methods section of the article. For studies not directly mentioning the abiotic (e.g., water) and biotic agents (animals), the fruit type of the identified experimental plant species was researched using Google and other sources. Lastly, SigmaPlot version 10.1 was used to analyze and make charts based on the extracted data.

### 4.4. Scope and Limitations

Only peer-reviewed, freely available publications written in English, at least indexed in Scopus, and published between 1990 and 2022 were included in the present systematic review. Other materials such as perspective papers, editorial notes, commentary, news articles, brochures or technical manuals were not considered in the present systematic review. The present SLR relies on the three most popular databases (i.e., ScienceDirect, PubMed, and Google Scholar) for peer-reviewed original articles for the identification of potentially eligible studies.

## 5. Conclusions

This systematic review summarizes the existing understanding of the projected effects of changing environments on the complex processes of seed dispersal across regions. Results revealed that global climate changes, by means of altering air temperature, rainfall, wind speed, the biophysical environment (e.g., habitat quality, fruit availability) and plant/seed traits, may negatively affect the seed dispersal patterns of vertebrate-dispersed plants, thereby influencing the fate of local and global biodiversity. The six groups of plants whose long-distance seed dispersal are seriously affected by changing environments include anemochorous, endozoochorous, epizoochorous, hydrochorous, myrmecochorous, and ornithochorous species. The present review also found that fruit/seed traits control the fate of seed dispersal depending on environmental conditions. Overall, the findings of the present systematic review provide a summary of evidence on how plants keep up with climate change as populations of seed dispersers decline. However, increased research investments in the less-studied seed dispersal vectors in megadiverse, hotspot countries are recommended to enhance understanding of how plants would keep up with climate change. Future research endeavors will also benefit significantly from investigations that advance interdisciplinary perspectives and collaboration to study seed dispersal processes across landscapes, plant growth forms, and taxa of dispersers amid climate change. Lastly, all the recommendations provided will be critical for evidence-based biodiversity conservation and informed policy formation in changing ecosystems.

## Figures and Tables

**Table 1:** The identified most frequently studied dispersal strategies by fruit type in the reviewed studies.

Dispersal Mode	Relative Count (%)	Fruit Type	Example Species
Anemochory	17.21	Achenes, capsule, winged seeds/samara	*Erigeron bonariensis*, *Pinus halepensis*, *Swietenia humilis*
Autochory/Ballochory	3.24	Dehiscent pod	*Pentaclethra macroloba*
Endozoochory	33.33	Capsule, drupe, fleshy, nuts,	*Tristerix corymbosus*, *Ficus carica*, *Duckeodendron cestroides*
Epizoochory	6.45	Achenes, drupe, dry dehiscent, fleshy, winged seeds/samara	*Choerospondias axillaris*, *Carapa procera*, *Heterosperma pinnatum*
Hydrochory/ichthyochory	9.68	Buoyant seeds, drupe	*Lolium perenne*, *Trifolium repens*, *Iris pseudocorus*
Myrmecochory	7.53	Achenes, capsule, fleshy	*Eucalyptus tetrodonta*, *Hepatica nobilis*, *Trillium undulatum*
Ornithochory	22.58	Capsule, drupe, fleshy, nuts,	*Prunus avium*, *Quercus serrata*, *Olea europaea*

**Table 2:** Seed dispersal-related traits of fruit/seeds of different plants in a changing environment.

Fruit/Seed Types	Examples	Adaptive Traits in a Changing Environment	
		Morpho-Structural Traits	Biochemical and Molecular Traits
Fleshy	Berry, drupe, hesperidium, pepo, pome	Soft pericarp, some have tough rind for edibility and long-distance seed dispersal; size and color	Nutrient and water content of mature fleshy fruit pulp for dietary requirements of frugivorous seed dispersers [16,17,18]; sugar, fat, and protein, antioxidants, minerals, and vitamins [19,20]; pigments and volatile compounds, cell wall modifications (softening) [21]
Dehiscent dry fruits	Capsule, silique, follicle, legume	Dry with single or multiple pod walls that facilitate fruit opening for seed dispersal	Programmed cell death (PCD) of pericarps; dead pericarps for long-term storage of nucleases, proteases, and chitinases [22]
Indehiscent dry fruits	Caryopsis, nut (acorn), samara, achene	Hard, dry, and dead pericarps for embryo protection and long-distance dispersal	Concentration of glucosinolates (GSLs) in seeds and pericarps that could explain dispersal strategies and dormancy of indehiscent fruits [23].

**Table 3:** The search terms used, and the initial results returned in the ScienceDirect, PubMed, and Google Scholar databases, as well as direct search.

Search Keywords	ScienceDirect	PubMed	Google Scholar	Direct Search	Total
“seed dispersal” AND “climate change”	1675	135	1700	21	3531
“spore dispersal” OR “propagule dispersal” AND “changing climate”	783	30	1110	10	1933
“plant spread” AND “climate”	280	931	3940	15	5151

**Table 4:** The criteria used for the extraction of information from the selected peer-reviewed original articles published during the last three decades.

Extraction Criteria	Information Considered and Justification
Publication year	Between 1990 and 2022, to obtain enough studies about seed dispersal in a changing environment.
2. Study site (country)	Global, to map the geographical distribution of studies and the trends of in studies about the reviewed topic.
3. Research topic	Keywords in the title, to provide insights into the research trends in seed dispersal-changing environment studies during the last three decades.
4. Experimental plant species	The plant species mentioned as the experimental plant species in the paper, to validate the dispersal modes of the species depending on their fruit types or plant traits.
5. Fruit types	Fruit types of the experimental plant species, to validate whether the dispersal modes/vectors match with fruit types.
6. Dispersal modes and vectors	Any mentioned mechanism/agent of seed dispersal, to determine which among the dispersal modes and vectors are well-studied.

## Data Availability

All the data used are already reflected in the article. Other relevant data may be available upon request from the authors.
